# Brachial plexus avulsion induced changes in gut microbiota promotes pain related anxiety-like behavior in mice

**DOI:** 10.3389/fneur.2023.1084494

**Published:** 2023-02-08

**Authors:** Jian-lei Zhang, Hang Xian, Rui Zhao, Ceng Luo, Rou-gang Xie, Tong Tian, Rui Cong

**Affiliations:** ^1^Department of Orthopedics, Xijing Hospital, Air Force Medical University, Xi'an, China; ^2^Department of Neurobiology, School of Basic Medicine, Air Force Medical University, Xi'an, China

**Keywords:** brachial plexus avulsion, microbiota-gut-brain axis, metabolites, anxiety, psychobiotics

## Abstract

**Introduction:**

Brachial plexus avulsion (BPA) injury develops frequent and intense neuropathic pain, involving in both peripheral and central nervous systems. The incidence of anxiety or depression caused by BPA-induced neuropathic pain is high, but the underlying mechanism remains unclear.

**Methods:**

We established a BPA mice model and assessed its negative emotions through behavioral tests. To further explore the role of the microbiota-gut-brain axis in the unique emotional behavior after BPA, we performed intestinal fecal 16s and metabolomics assays. Psychobiotics (PB) supplementation was administered to BPA mice to check the probiotics effects on BPA-induced anxiety behaviors.

**Results:**

Pain related anxiety-like behavior was observed at the early stage after BPA (7 days), while no depression-like behavior was detected. Intriguingly, gut microbiota diversity was increased in BPA mice, and the most abundant probiotics, Lactobacillus, showed obvious changes. Lactobacillus_reuteri was significantly decreased in BPA mice. Metabolomics analysis showed that Lactobacillus_reuteri-related bile acid pathway and some neurotransmitter amino acids were significantly altered. Further PB (dominated by Lactobacillus_reuteri) supplementation could significantly relieve BPA-induced anxiety-like behaviors in mice.

**Conclusion:**

Our study suggests that pathological neuralgia after BPA could alter intestinal microbiota diversity, especially Lactobacillus, and the changes in neurotransmitter amino acid metabolites may be the key reason for the onset of anxiety-like behaviors in BPA mice.

## 1. Introduction

Brachial plexus injury (BPI) is a common and special type of peripheral nerve injury, which usually occurs in traffic accidents, especially motorcycles (1). BPI is often accompanied by severe pain complications called neuropathic pain (NPP), which is characterized by spontaneous pain, hyperalgesia, and hyperalgesia. The incidence of NPP is high in the patients with BPI. Around 30–80% patients within brachial plexus avulsion (BPA) show symptoms of NPP (2, 3). BPA is a mixed injury involving both peripheral and central nervous mechanisms (4). BPA causes immediate pain which gets rapid onset after trauma; along with the progression of long-term neuropathy, this pain may be observed away from the lesions (5). Nowadays, the research on BPA mainly focused on nerve transplantation procedures, which ignores BPA-induced pain and related comorbidities like emotional and/or cognitive impairments that largely worsen the quality of patients' life (6). Previous reports have revealed that the incidence of negative stress responses, including anxiety and depression, of patients with BPA could reach up to 30–50% (7). A follow-up study of 415 patients with BPA found that 81.20% of them appeared anxiety or depression (8). Another clinical trial of BPA patients also showed that even with active surgical intervention, there were still 76% of patients suffered by frequent pain and 18.8% of them accompanied with depression or anxiety (9). Therefore, BPA induced neuropathic pain and related negative emotions should get more attention.

The impact of symbiotic microbiota on various host functions has been increasingly recognized over the past decade. It is well-studied that gut microbes influence many biological processes including immunity, metabolism, and central nervous system (CNS) (10–12). The effects of gut microbiota on NPP animal models have also been characterized (13, 14). Extensive studies have shown that the microbiota can directly or indirectly influence brain homeostasis through affecting anxiety and the occurrence of neurological diseases were related to nerve conduction dysfunction in animal models (15). Therapeutic intervention with probiotic can affect the emotional behaviors of depressive mice and alleviate depression in patients (16, 17). In this study, to explore whether gut microbiota also play roles in the negative emotions caused by BPA, we investigated the changes of behaviors, gut microbes, and metabolites in BPA mice through bioinformatics analysis of 16s sequencing and metabolomics, and also provided supporting evidence for the therapeutic intervention with intestinal probiotics.

## 2. Methods and materials

### 2.1. Establishment of BPA mice model

**Figure 1:**
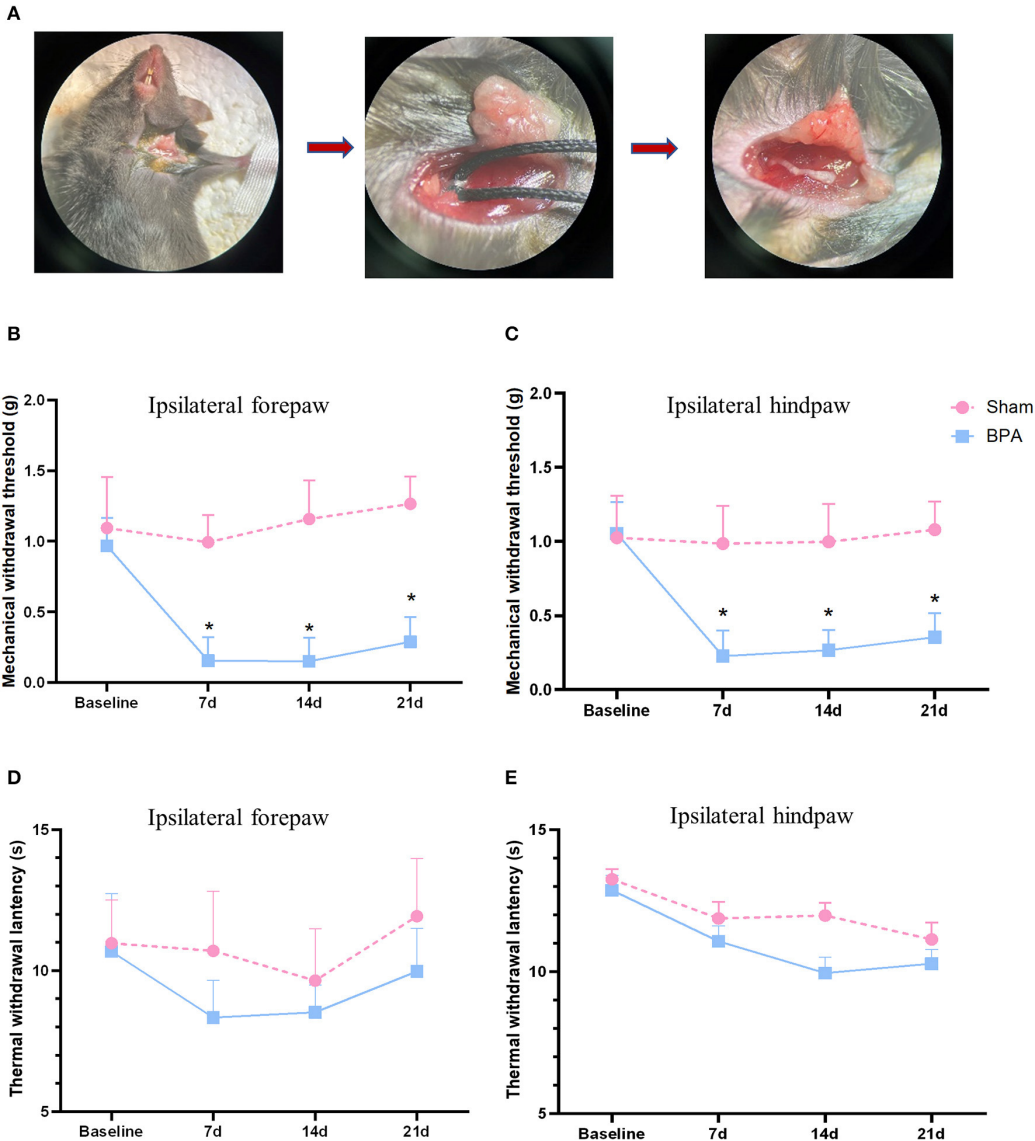
BPA modeling and pain behavior detection over time. **(A)** Schematic diagram of BPA operation: from brachial plexus dissociate the C7 nerve separation to nerve avulsion. **(B)** PMWT results of the operative limb 1 day before and 7, 14, and 21 days after modeling. **(C)** PMWT results of the ipsilateral hindlimb of sham and BPA mice. **(D)** No significant difference of TWL in the operative limb between sham and BPA mice at 1 day before and 7, 14, and 21 days after modeling. **(E)** TWL results of the ipsilateral hindlimb of the two group at each time point. *N* = 10 per group. *P < 0.05 vs. sham at the same time point. Unpaired *T*-test was used for data analysis. BPA, brachial plexus avulsion; PMWT, paw mechanical withdrawal threshold; TWL, thermal withdrawal latency.

Adult female C57BL/6 mice (6–8 weeks old, 20–25 g) were randomly divided into sham and BPA group (*n* = 10 in each). The mice were housed in the specific pathogen-free laboratory of Animal Center of Air Force Medical University. During modeling, the mice were anesthetized with 1% sodium pentobarbital (45 mg/kg) and fixed on the operating table. The upper limbs were stretched, and the left limb was chosen to carry out a transverse incision (0.8–1.2 cm) at 0.3–0.5 cm under the collarbone. Brachial plexus was separated and the C7 nerve root was exposed. The 2–0 silk thread was used to wrap the C7 nerve, then the avulsion injury was performed through the stretch of the thread under a constant speed. The visibility of the corresponding nerve root and dorsal root body are considered as successful avulsion (Figure 1A). After avulsion, the stump was put back to the muscle space between pectoralis major and minor muscles, followed by incision suture using 3–0 silk thread. The mice were reared in cages after waking up. Mice in sham group underwent same operation to expose the brachial plexus without any further injury.

### 2.2. Behavioral testing

#### 2.2.1. Pain behavior detection

The basal and threshold value of pain behavior on the left forepaw and left hindpaw were measured on 1 day before and 7, 14, and 21 days after BPA modeling. Behavioral parameters of paw mechanical withdrawal threshold (PMWT) and thermal withdrawal latency (TWL) were assessed.

To evaluate PMWT, one black plastic box with a lid was placed on a rack with a metal mesh (0.3 × 0.3 cm) at the bottom, and the test was carried out after 0.5–1 h adaptation. Von Frey filaments of different grams were used to detect the skin sensation of the mice planta pedis. The planta was stimulated 5 times at an interval of 3 min, and mice showed a positive selection when they lifted or licked their paws. The gram thresholds were recorded and the average of them was calculated.

To evaluate TWL, the black plastic box with a lid was placed on a glass plate (35°C). Environment adaptation was conducted 0.5–1 h before testing. The thermal radiation intensity and the irradiation time were set in advance to prevent skin burns caused by prolonged irradiation to the mice planta pedis. The maximum irradiation time was set as 20 s. The mice planta pedis was irradiated with pre-set thermal radiation intensity. The irradiation was stopped immediately and the time was recorded when the mice retracted, lifted, shook, or licked their paws. After five repetitions, the average time was calculated as the TWL value.

#### 2.2.2. Motor ability detection

The beam walking test (BWT) and Rota-Rod test were conducted to detect the effects of BPA on the murine motor function. For BWT, one side of the balance beam was placed in a black square box, and the mouse was placed on the other side. The time and number of skids for mice entering the black square box through the balance beam were recorded within 60s. The test included training period and testing period. If the mouse failed to reach the box, then the time was recorded as 60s. Rota-Rod test also had pre-training and testing periods. During testing, the animal was placed on the rotating rod. Once fell off the rod, the mice were put back immediately. The latency time for falling and rotation speed during falling were recorded. After three repetitions, the average delay time and rotation speed were calculated.

#### 2.2.3. Behavioral testing for anxiety and depression

Open field test (OFT): OFT was performed to detect the animal autonomous behavior, exploratory behavior, and tension in a new environment. The open field box (50 × 50 × 35 cm, L × W × H) was placed with a camera directly above the box connecting to the computer. EthoVision XT behavioral video recording software was used to record the animal behavior during experiments. The mouse was placed in the center of the open filed with the back to the researcher. Moving trajectory was recorded for 10 min, and the movement distance, speed, and residence time in the central area were analyzed.

Elevated plus-maze test (EPM): The EPM equipment consisted of two open arms (30 × 7 cm) and two closed arms (30 × 7 × 15 cm) with a height of 60 cm from the ground. During experiments, all mice were placed in the same position at the center of the maze with their head to the open arms. The entry times of open and closed arms and the duration within each arm in 5 min were recorded.

Tail suspension test (TST): The rear 1/3 of the tail of the mouse was hung on a support with the head 15 cm away from the table. Six minutes later, the immobile time of mice was recorded and analyzed using Smart v3.0 animal behavior analysis software.

Forced swimming test (FST): The mouse was put into a transparent cylinder (10 × 18 cm, Diameter × H) for 6 min that had been filled with water at 25 ± 1°C. The animal activity in the water was recorded automatically by Smart v3.0 animal behavior analysis software. The immobile duration of mice was analyzed.

Each group of 10 mice was independent of the different behavioral tests at each time point.

### 2.3. Gut microbiota 16s sequencing of BPA mice

Fecal samples (150–200 mg) from ileocecal sections were collected on 14 days after BPA modeling. Each group contained 8 mice at the beginning, and only 5 samples in the sham and 7 samples in the BPA group passed quality detection and go forward following sequencing and analysis. HiPure Stool DNA Kits (#D3141, Megan Biotechnology Co., LTD., Guangzhou, China) was used to extract fecal DNAs, that were then detected by Nanorop 2000 spectrophotometer (Thermo Fisher Scientific, Waltham, MA, USA), agarose gel electrophoresis apparatus DYY-6C (BJLiuYi, Beijing, China), and gel imaging system Tanon-2500 (Shanghai Tianeng Technology Co., LTD., China). The second round pf PCR was performed after quality qualified. AMPure XP Beads were used to purify the amplification products, and ABI StepOnePlus Real-Time PCR System (Life Technologies, Carlsbad, CA, USA) was used for quantification. Sequencing was performed according to the PE250 mode pooling of Novaseq 6000. For 16s sequencing data analysis, raw reads were firstly filtered by the rules: reads contained >10% of unknown nucleotides and/or <50% of bases with quality >20. Then, filtered reads were assembled into raw tags through FLASH. After raw tag filtering, operational taxonomic units (OTUs) were obtained *via* clean tag clustering by UPARSE pipeline and chimera removal was conducted to get effective tags for further analysis.

### 2.4. Sequencing of intestinal metabolites in BPA mice

The cultural medium from the mice fecal samples was removed. LC-MS/MS analyses were performed using an UHPLC system (1,290, Agilent Technologies, Santa Clara, CA, USA) with a UPLC HSS T3 column coupled to a Q Exactive mass spectrometer (Orbitrap MS, Thermo Fisher Scientific). The mobile phase A was 0.1% formic acid in water for positive mode, and 5 mmol/L ammonium acetate in water for negative mode, and the mobile phase B was acetonitrile. The injection volume was 2 μL. The QE mass spectrometer was used because its ability to acquire MS/MS spectra on an information-dependent basis (IDA) during an LC/MS experiment. In this mode, the acquisition software (Xcalibur 4.0.27, Thermo Fisher Scientific) continuously evaluates the full scan survey MS data as it collects and triggers the acquisition of MS/MS spectra depending on preselected criteria. ESI source conditions were set as follows: sheath gas flow rate was 45 Arb, aux gas flow rate was 15 Arb, capillary temperature was 320°C, full MS resolution was 70,000, MS/MS resolution was 17,500, collision energy was 20/40/60 eV in the NCE model, spray voltage was 3.8 (+) or −3.1 kV (–). Through bioinformatics analysis, potential differentially expressed metabolites were identified according to specific screening conditions (VIP > 1 and *P* < 0.05). Metabolomic profiling was performed in collaboration with Guangzhou Genedenovo Biotechnology Co., Ltd. (Guangzhou, China).

### 2.5. Association analysis of the intestinal microbiota and metabolites bioinformatics in BPA mice

Pearson correlation coefficient of microbiota and metabolomic datasets between every level were calculated in R (version 3.5.1). The correlation heatmap was generated using pheatmap package in R. The network analysis was performed using igraph package in R.

### 2.6. Effects of psychobiotics (PB) supplementation on BPA mice behavior

The mice were randomly divided into sham, BPA, and BPA + PB groups (*n* = 10 per group). Mice in the BPA + PB group were intragastrically administrated with PB every day after BPA modeling. The PB used was purchased from Jiangnan University containing Bifidobacterium bifidum F-35, Bifidobacterium longum CCFM729, Lactobacillus plantarum CCFM639, Lactobacillus acidophilus CCFM137, Lactobacillus casei CN1566, Lactobacillus reuteri DSM17938, and Lactobacillus rhamnosus CCFM10281 (0.02 mg/kg). Mice in sham and BPA groups were intragastrically administrated with galactose oligosaccharide (20.2%). Pain behavior detection, OFT, and EPM tests were carried out 1 day before and 14 days after operation.

### 2.7. Statistical analysis

A total of 126 mice were used and randomly divided into each group for behavior tests (*n* = 10 per group) and 16s sequencing (*n* = 8 per group) in this study. Analyzed data were expressed as mean ± standard deviation (SD), and statistically analyzed by unpaired *T*-test within two groups and ordinary one-way ANOVA within three groups using GraphPad Prism 8.0 software. Besides, *p*-value < 0.05 was considered statistically significant.

## 3. Results

### 3.1. BPA could induce stable mechanical allodynia of the affected forepaw

After successful procedure of avulsion injury, the single C7 nerve root could be observed (Figure 1A). Mechanical threshold of the affected forepaw and ipsilateral hindpaw decreased at 7 days after BPA and lasted until 21 days, which had significant differences compared to sham group (Figures 1B, C). While, no obvious difference was observed in TWL tests between the two groups of the ipsilateral forepaw and ipsilateral hindpaw (Figures 1D, E). These results indicated that this single C7 root avulsion model could mimic the neuropathic pain state like BPA patients which fulfills the needs of the following investigations.

### 3.2. Comorbidity of pain related anxiety-like behavior occurred after BPA happens

**Figure 2:**
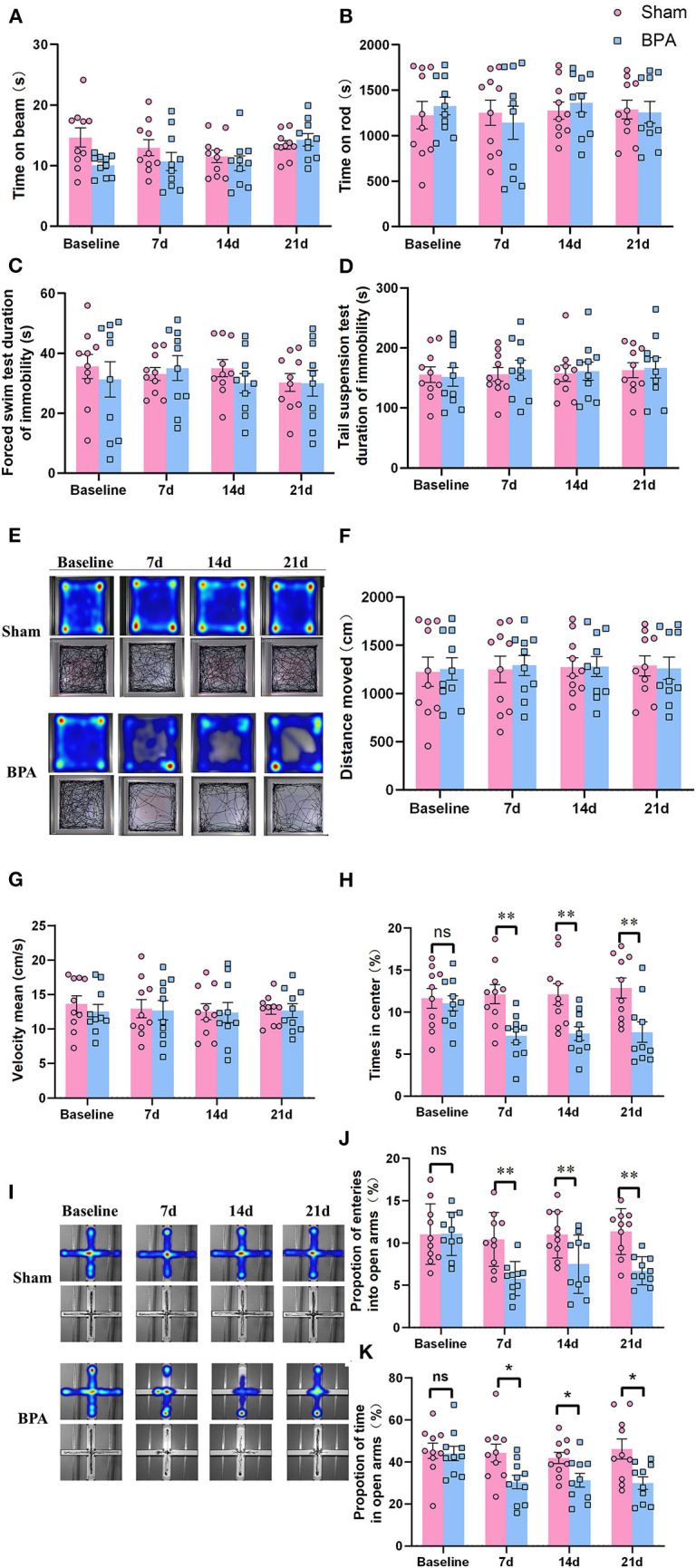
Detection of depression and anxiety-like behaviors in BPA mice over time. **(A)** BWT detection showed no difference of the time on the beam at each time point. **(B)** Rota-Rod test showed no difference of the time on the rod at each time point. **(C)** FST showed no difference of the immobility duration at each time point. **(D)** TST shows no difference of the immobility duration at each time point. **(E)** OFT trajectory diagram in the two groups over time. **(F)** The movement distance from OFT test between the two groups at each time point. **(G)** The movement speed from OFT test between the two groups at each time point. **(H)** The central area residence time from OFT test between the two groups at each time point. **(I)** EPM trajectory diagram in the two groups. (J) EPM: The entering times of the open arms at each time point. **(K)** EPM: The open arm residence time at each time point. *N* = 10 per group. Ns = no statistical significance, *P < 0.05, **P < 0.01 vs. sham. Unpaired *T*-test was used for data analysis. BPA, brachial plexus avulsion; BWT, beam walking test; FST, forced swimming test; TST, tail suspension test; OFT, open field test; EPM, elevated plus-maze test.

BWT detection showed that there was no significant difference in balance ability, muscle strength, and motor coordination between sham and BPA mice at 1 day before and 7, 14, and 21 days after modeling (Figure 2A). Similarly, in Rota-Rod test, no difference of time on rod was observed between sham and BPA mice at each time point (Figure 2B). The immobile duration from FST and TST results indicated that mice in BPA group had no significant depression-like behavior before and after modeling compared to the sham group (Figures 2C, D). In addition, the trajectory diagram of OFT experiments clearly showed a decrease of the residence track in the center area at 7, 14, and 21 days after BPA modeling (Figure 2E). Statistical analysis showed no significant difference in the movement distance and speed of mice between the two groups (Figures 2F, G), but the residence time at the center area was significantly decreased at 7, 14, and 21 days after BPA compared with sham group (Figure 2H). Moreover, EPM trajectory diagram showed that that the open arm entering frequency was reduced after BPA modeling (Figure 2I). Meanwhile, the entry times of open arms and the duration were significantly reduced in BPA group compared with the sham (Figures 2J, K). The above results showed single root avulsion won't affect the motor function of the BPA mice, and the pain related anxiety-like behavior occurred after BPA.

### 3.3. BPA altered gut microbiota composition

**Figure 3:**
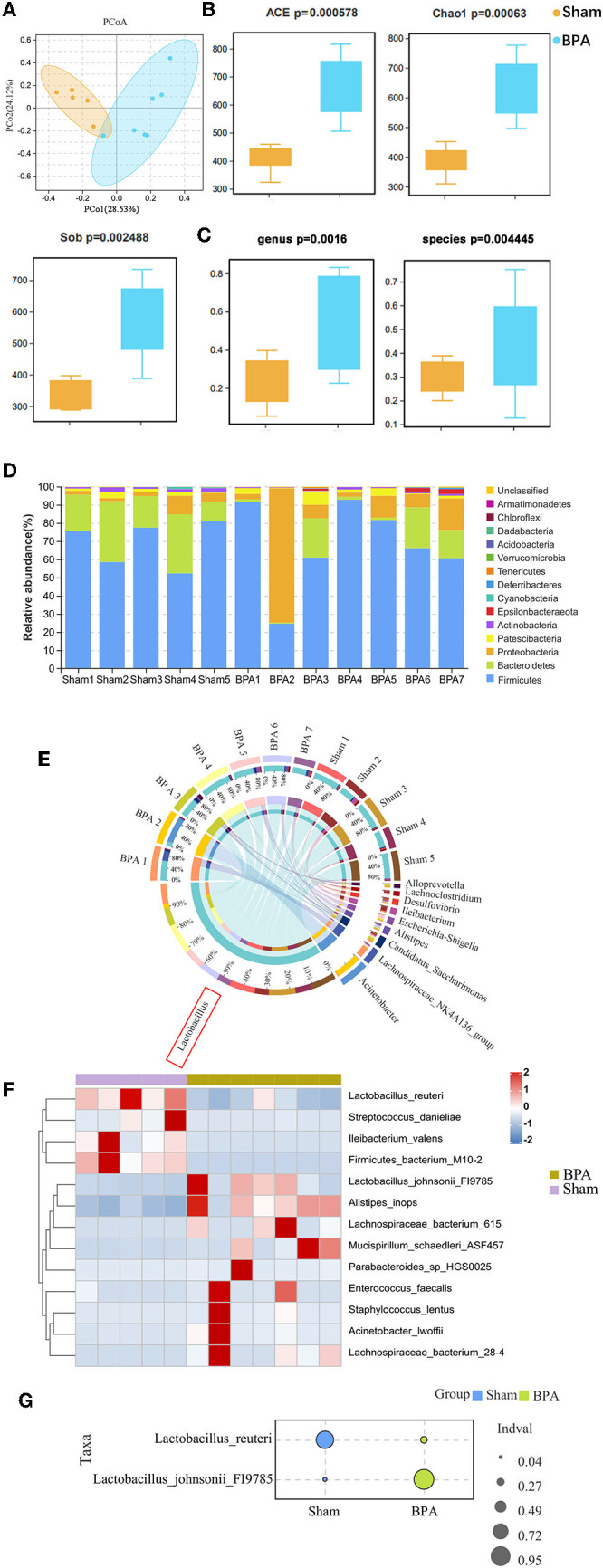
Gut microbiota 16s sequencing of BPA mice. **(A)** PcoA plot of all samples. **(B)** α diversity of ACE, Chao1, and Sob indices of the gut microbiota in the two group **(C)** β diversity analysis of the samples in the two groups at the genus and species level. **(D)** Relative abundance of gut microbiota at the phylum levels in the two groups. **(E)** Distribution circle map of 16s species composition. **(F)** Heat map showing that indicator species analysis between the groups. **(G)** Indicator value analysis of 16s indicator species, and Lactobacillus_reuteri and Lactobacillus_johnsonii with the most significant differences between groups were shown. *N* = 5 in BPA group and *N* = 7 in sham group. BPA, brachial plexus avulsion; PcoA, principal coordinates analysis.

To investigate changes in gut microbiota induced by BPA-caused NPP in mice, 16s rDNA amplicons from fecal samples of sham (*n* = 5) and BPA groups (*n* = 7) were sequenced. Principal coordinates analysis (PcoA) of groups indicated that in general, the species community structure was similar within the two groups but distinct between them (Figure 3A). The α-diversity of microbiota between sham and BPA samples was compared by ACE, chao1, and Sob indices. Alpha-diversity mainly reveals species richness by counting the number of species or OTUs. Results from all three α-diversity indices showed that compared to the sham, samples in BPA had significantly increased α-diversity, indicating elevated species richness (Figure 3B). Another β diversity analysis informs the diversity of microbiota. Results of the β diversity of the samples in the BPA groups were also significantly higher than that in the sham, at both genus and species levels (Figure 3C). Hence, BPA could increase the richness and diversity of intestinal flora. Moreover, relative abundance of gut microbiota at the phylum level in the two groups was shown. The three largest bacterial phyla were Firmicutes, Bacteroidetes, and Proteobacteria (Figure 3D). Moreover, species composition distribution showed that Lactobacillus genera had the most obvious change (Figure 3E). We further analyzed the significant flora changes at the species level, and 13 significantly-altered species were shown (Figure 3F). Among them, Lactobacillus_reuteri and Lactobacillus_johnsonii were the two species with the most significantly different indicator values after BPA through the indicator value analysis *via* R package (Figure 3G).

### 3.4. BPA altered gut metabolites

**Figure 4:**
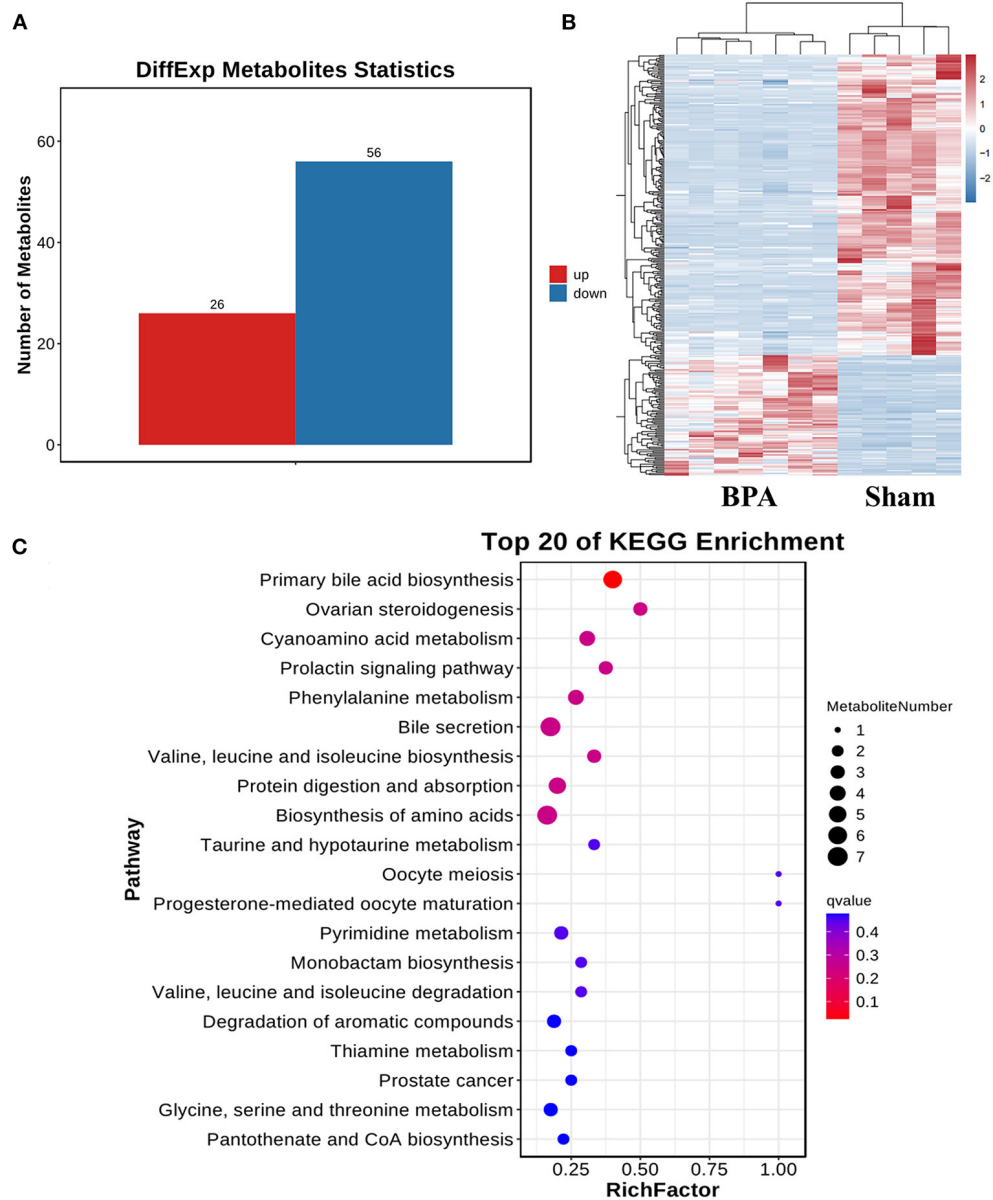
Gut bacteria metabolites sequencing analysis of sham and BPA mice. **(A)** Differentially expressed metabolites in BPA groups compared to the sham group. **(B)** Cluster heat map of the differentially expressed metabolites between the two groups. **(C)** KEGG functional enrichment analysis of the differentially expressed metabolites.

To identify BPA-induced metabolic disorders, metabolomics analysis was performed between sham (*n* = 5) and BPA (*n* = 7) groups. Compared with sham, 82 differentially expressed metabolites were identified in BPA, including 26 up-regulated and 56 down-regulated metabolites (Figure 4A). Clustering heat maps were used to visualize the significantly differential-expressed metabolites between the two groups (Figure 4B). Further KEGG functional enrichment analysis of these differentially expressed metabolites showed that differentially expressed metabolites mainly participated in Pantothenate and CoA biosynthesis, Primary bile acid biosynthesis, Bile Hair's-gain, Glycine, serine and threonine metabolism (Figure 4C).

### 3.5. Correlation analysis between intestinal microbiota and metabolites in BPA mice

**Figure 5:**
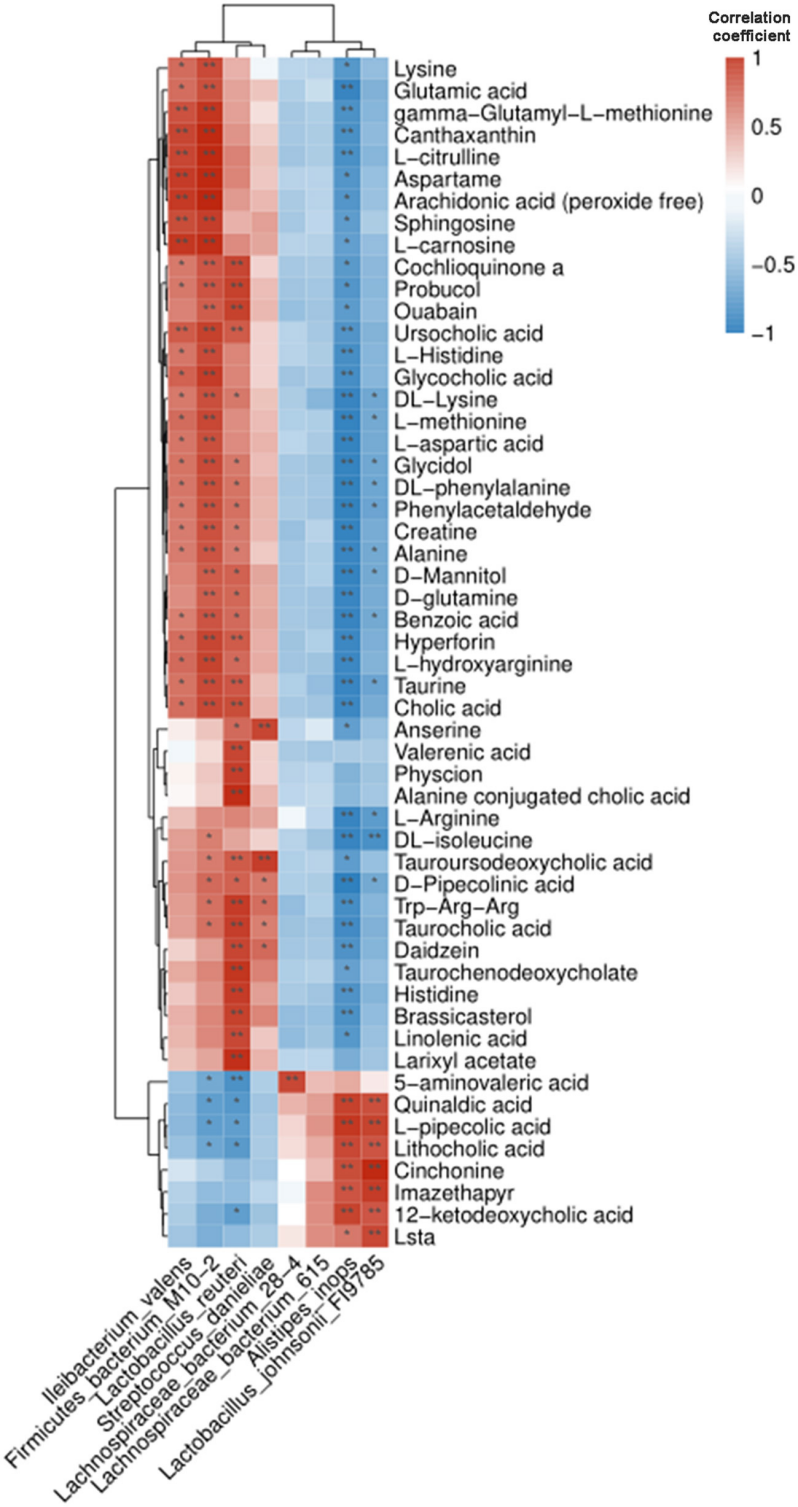
Pearson correlation analysis of intestinal microbiota and metabolites in BPA mice. The cluster heat map showed their correlation. Blue and red represented the negative and positive correlation, respectively. *P < 0.05, **P < 0.01.

To further investigate the functional significance of metabolic dysfunction caused by intestinal microbiota disorder in BPA mice, Pearson correlation analysis was applied to find the association between 8 abundant intestinal bacteria at the genus level and 54 differentially expressed metabolites. The cluster heat map visualized the Pearson correlation coefficient (Figure 5).

### 3.6. PB supplementation could relieve anxiety-like behaviors in BPA mice

**Figure 6:**
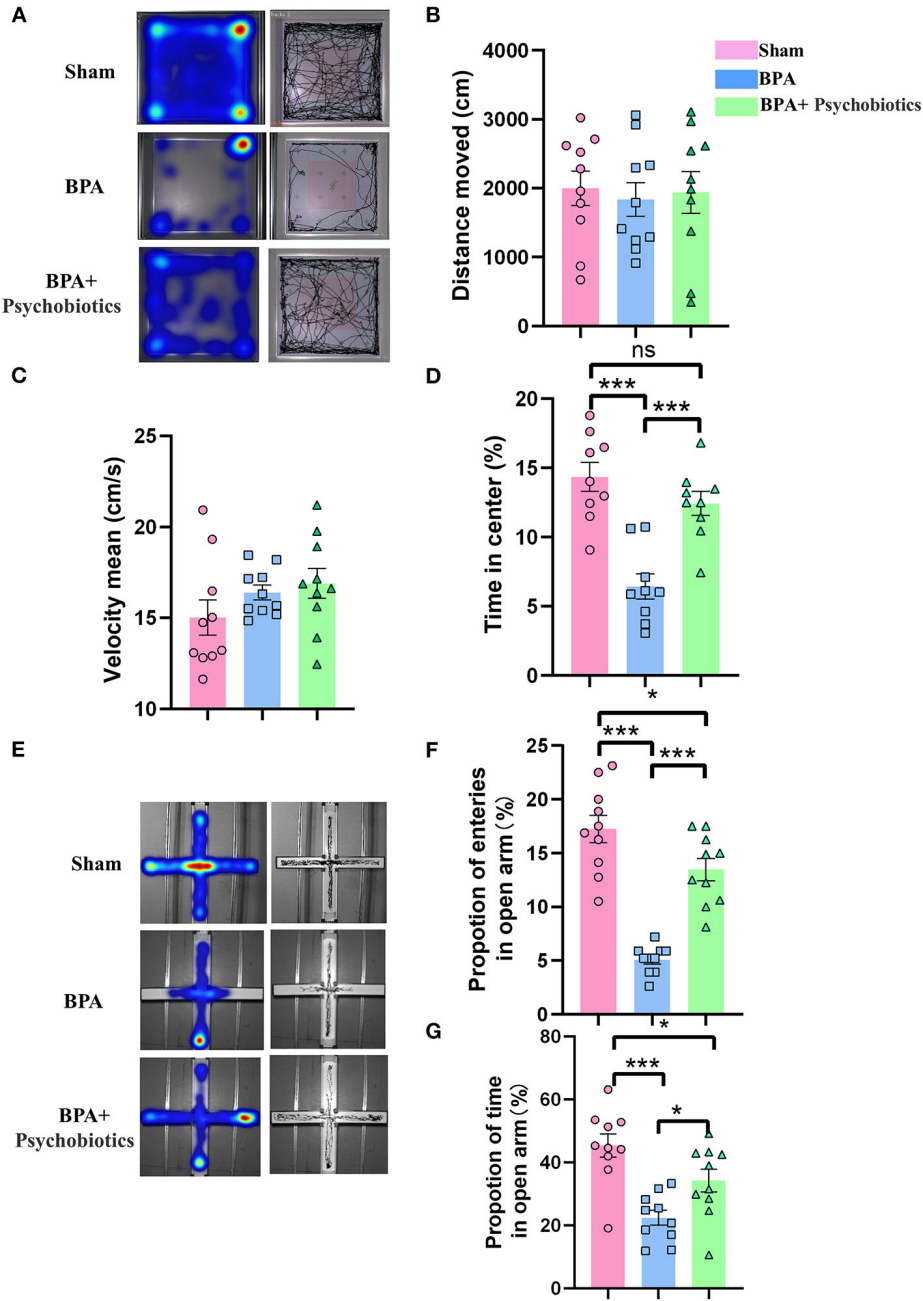
Effects of PB supplementation on anxiety-like behaviors in BPA mice. **(A)** OFT trajectory diagram. **(B)** The movement distance from OFT tests among the three groups. **(C)** The movement speed from OFT tests among the three groups. **(D)** The residence time in the central area from OFT among groups **(E)** EPM trajectory diagram. **(F)** EPM: The proportion of open arm entry times in the three groups. **(G)** EPM: The open arm residence time among groups. *N* = 10 per group. Ns = no statistical significance, *P < 0.05, ***P < 0.001. Ordinary one-way ANOVA was used for data analysis. BPA, brachial plexus avulsion; PB, psychobiotics; OFT, open field test; EPM, elevated plus-maze test.

BPA mice were then intragastrically administrated with PB every day until 14 days after modeling, followed by pain detection, OFT, and EPM testing. Pain detection showed that PB treatment significantly attenuate the mechanical allodynia of BPA mice (Supplementary Figure 1). OFT results showed that there was no significant difference in the movement distance and speed among the three groups (Figures 6A–C), while the reduced residence time in the center area of the BPA group was significantly increased by PB supplementation (Figure 6D). EPM results showed that BPA decreased the frequency of entering the open arm and the residence time of the open arm compared with the sham, which were significantly reversed after PB supplementation (Figures 6E–G). These results showed that probiotics could significantly relieve pain related anxiety-like behavior after BPA.

## 4. Discussion

Our study found the evidence of the participation of microbiota-gut-brain axis in the pathological process of BPA induced pain related anxiety-like behavior in mice. 16s rDNA amplification of intestinal microbiota showed that BPA significantly affected the diversity of intestinal microbiota, and a variety of mental probiotics were significantly decreased. The differentially expressed metabolites were involved in the metabolism and synthesis of various amino acids in response to BPA stress in the gut. Some of the metabolites as neurotransmitters or neurotransmitter precursors are significantly different, and most of the intestinal flora related to these metabolites belong to the category of neuroprobiotics. Therefore, we considered that the onset of BPA-induced anxiety-like behaviors may be related to the altered gut microbiota, especially the reduction of psychogenic probiotics. Supplementation with related probiotics significantly attenuated anxiety-like behaviors in BPA mice, which supported that the occurrence of anxiety-like behaviors after BPA is close to the changes of related microbiota and metabolites.

Nerve injury-induced acute pain is a warning mechanism of the human body, which prevents further tissue damage (18). Due to the great changes in peripheral nervous system (PNS) and CNS after nerve injury, nociceptive sensitization happens, which leads to intestinal microbiota disorders, and in turn affects the PNS and CNS through metabolites after adapting to the pain stimuli. This microbiota-gut-brain interaction involves in the regulation of neuroimmunity, host metabolism, neurotransmitter system, and vague nerve (19). Peripheral and central sensitization are crucial issues during the chronicity of pain, and are also the main reasons for the comorbidities including anxiety, depression, etc. About 30–60% of patients with chronic pain suffered by depression, and the prevalence of suicide consciousness is 32% (20). Even with active surgical treatment, the incidence of anxiety or depression in BPI patients is still higher than 18.8% (9), and in BPA patients is as high as 81.2% (8). In order to explore the underlying mechanisms, we constructed a BPA mouse model according to the operation process described by Xian et al. (21). The results showed that this model could mimic the pain state like BPA patients and single C7 nerve root avulsion had little effect on motor ability of mice. It is the pain related anxiety-like behaviors was observed at 7, 14, and 21 days after BPA but no depression-like behavior. Compared with previously reported results in chronic constriction injury (22) and partial sciatic nerve ligation neuralgia models (23), the onset of anxiety-like behaviors in BPA mice is earlier, the mechanism under this difference still worth deep mining.

In order to explore the effects of BPA on intestinal microbiota, we conducted 16s sequencing of intestinal microbiota of BPA mice to explore the correlation between microbiota and BPA induced pain related anxiety-like behaviors. Sequencing results found that the α and β diversity of the microbiota of BPA mice increased significantly, indicating the changes of species richness and diversity after BPA. The most obvious change was Lactobacillus. Lactobacillus is the most abundant probiotic bacteria in the gut, which maintains many functions of the body. Studies have found that Lactobacillus is an important member of the microorganism of the NPP mouse (24), and it has obvious changes both in acute and chronic neuralgia (25). Among the 13 species with the significant changes, Lactobacillus_reuteri showed significantly decreased indicator value after BPA, which may be related to the intestinal stress on pain, because Lactobacillus_reuteri can reduce the pain stimulation caused by nerve discharge (26). Lactobacillus increases short chain fatty acid (SCFA) metabolites, such as acetate, propionate, and butyrate, which are important regulators in the synthesis and degradation of neurotransmitters. Moreover, the colonization of Lactobacillus rhamnosus is beneficial to reduce anxiety (27). Therefore, we speculate that the reduction of Lactobacillus abundance induced by BPA may regulate the microbial-gut-brain axis through altering metabolites.

To investigate the changes of intestinal metabolites, we performed untargeted metabolomics of intestinal feces. The expression of 26 metabolites increased and 56 metabolites decreased in BPA mice. KEGG enrichment pathways of differentially expressed metabolites mainly focused on Pantothenate and CoA biosynthesis, Primary bile acid biosynthesis, Bile hair development, Glycine, serine and threonine metabolism. These pathways are related to the abnormal metabolism of bile acid. Intestinal microbiota synthesizes hundreds of metabolites that affect host physiology, among which the most abundant metabolites are secondary bile acids (28). Primary bile acids produced by liver are further synthesized by intestinal microbiota to about 20 types of bile acids (29). Bile acids regulate the neurotransmitter receptor functions, like muscarinic acetylcholine receptor and γ-aminobutyric acid receptor. Bile acids can also be protective against neurodegeneration (30). Another metabolite that was significantly elevated was glutamate, a key excitatory neurotransmitter for emotion regulation (31). The apparent rise in glutamate expression may be the body's response to painful stimuli. However, further studies are needed to determine whether these metabolites are related to the dysregulation of microbiota.

We further investigated the association between BPA microbiota and corresponding metabolites. We found that Alistipes_inops, Lactobacillus_johnsonii_FI9785, Lactobacillus_reuteri, and Firmicutes_bacterium were correlated with the expression of a large number of metabolites, including γ-aminobutyric acid (GABA), anandamide (AEA), and amino acid neurotransmitters. An interesting phenomenon was found that Lactobacillus_reuteri was positively correlated with AEA. AEA is closely related to the body's endogenous cannabinoid system (eCBS) (32). eCBS have pleiotropic functions *in vivo*. It plays a key role in the development of energy homeostasis and metabolic disorders and is a mediator of the relationship between the gut microbiota and host metabolism. These neurotransmitter amino acid changes were associated with four microbiotas, two of which were Lactobacillus, suggesting that the related metabolite changes caused by Lactobacillus microbiota may be a key factor in BPA-induced anxiety-like behavior. Therefore, we supplemented PB in BPA mice to test its effect on anxiety. PB is a probitic strain to affect gut-brain axis, and has been reported to improve microbiota and alleviate some symptoms of CNS disorders (33). The results showed anxiety-like behaviors of BPA mice were significantly relieved after PB supplementation. Therefore, we confirmed that the decrease of intestinal microbiota diversity caused by BPA, especially the changes of neurotransmitter amino acid metabolites caused by altered Lactobacillus diversity, may be the key causes of anxiety-like behavior.

**Figure 7:**
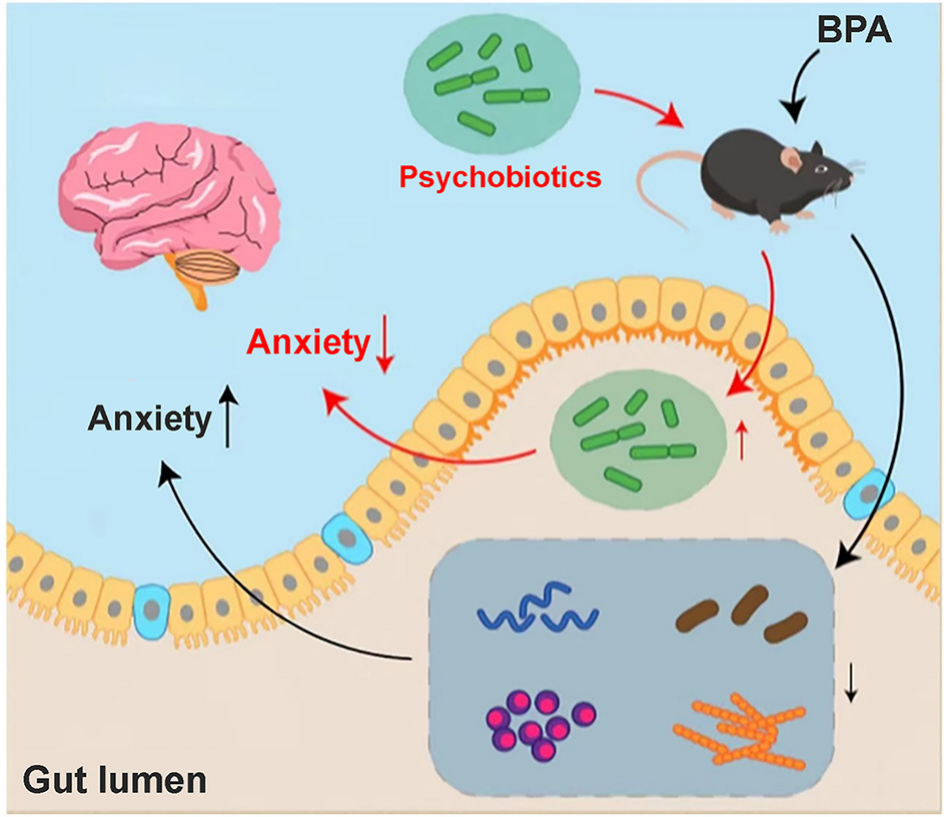
Hypothesis mechanism diagram of BPA mice. Anxiety-like behavior appeared in the BPA mice, which may be related to the disturbance of intestinal flora and the changes in metabolites after BPA. Through sequencing, we found that a variety of microbiota and its related metabolites involved in the anxiety inhibition decreased significantly, especially Lactobacillus_reuteri. The anxiety-like behavior after BPA can be significantly improved by the addition of intestinal psychoprobiotics, but the specific mechanism of brain-gut axis regulation still needs to be further explored.

In the current study, we found BPA could induce pain and related anxiety-like behavior in mice, and the participation of microbiota-gut-brain axis may be the critical point during this pathological process (Figure 7). BPA causes significant intestinal microbiota disorder and altered metabolites. Regulation of the microbiota and metabolites maybe the key point to cure this comorbidity induced by BPA. While, this is just a preliminary research, and what specific kind of metabolite affects the brain through microbiota-gut-brain axis remains unclear. As the influence of the microbiota-gut-brain axis is mutual, how the CNS regulates the corresponding neurotransmitters after receiving the signals of peripheral nervous system injury and the mutual feedback mechanism between the CNS and intestinal flora are still worth further explorations

## Data availability statement

The data presented in the study are deposited in the NCBI repository, accession number PRJNA899015.

## Ethics statement

The animal study was reviewed and approved by the Fourth Military Medical University Laboratory Animal Center of Welfare and Ethics Committee.

## Author contributions

J-lZ and RC proposed the idea and drafted the manuscript. J-lZ and HX performed animal models, behavioral tests, and data analysis. RZ and CL performed data analysis. R-gX and TT assisted figures editing. RC was responsible for supervising. All authors contributed to the article and approved the submitted version.
