# Supplementary figures and images for: Brachial plexus avulsion induced changes in gut microbiota promotes pain related anxiety-like behavior in mice
Source: Front Neurol. 2023 Feb 8;14:1084494. doi: 10.3389/fneur.2023.1084494 (PMC9944865; doi:10.3389/fneur.2023.1084494)

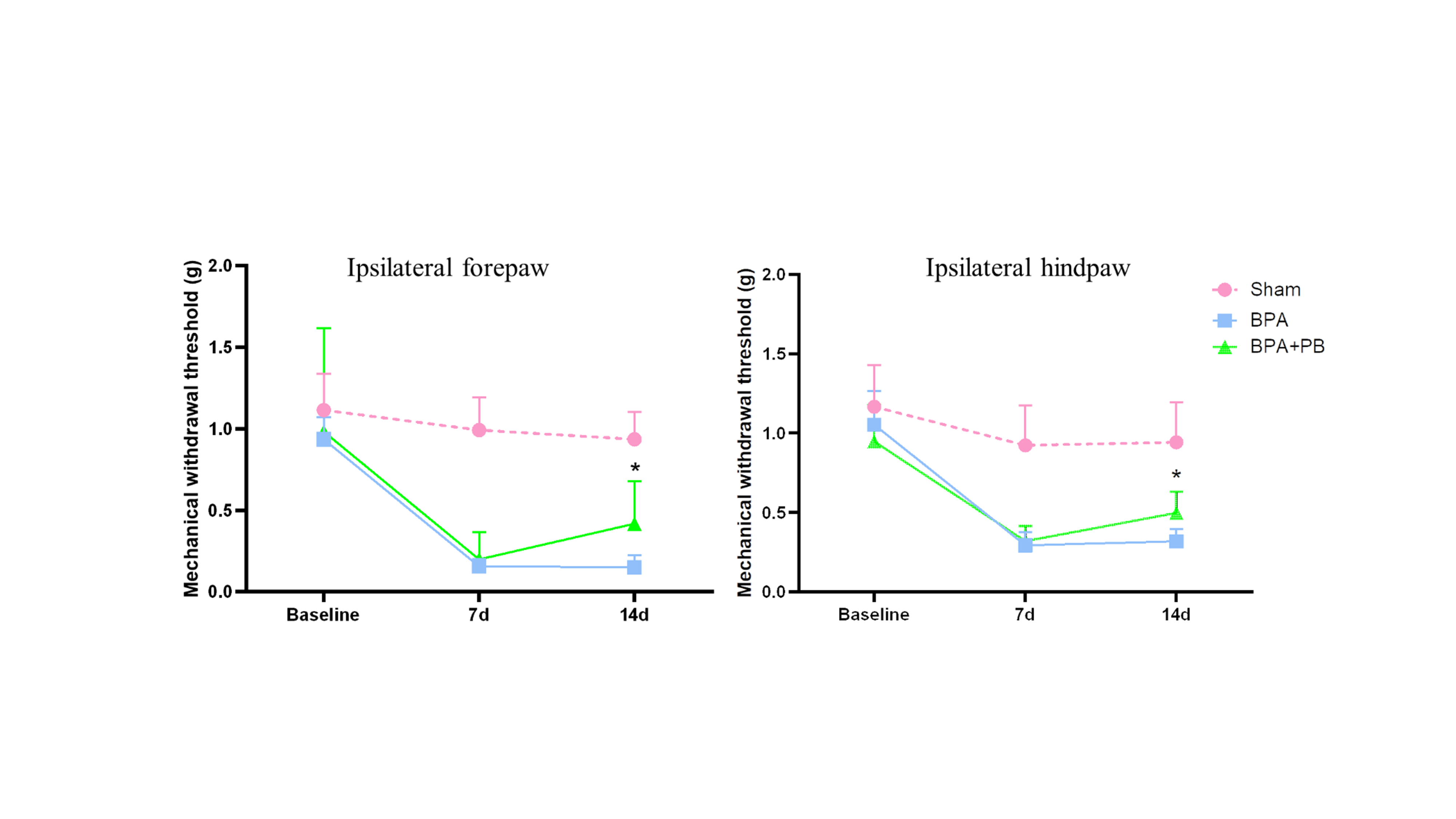

Supplement: Supplementary file 1 [file Image_1.TIF]
